# Expression of *Arf* Tumor Suppressor in Spermatogonia Facilitates Meiotic Progression in Male Germ Cells

**DOI:** 10.1371/journal.pgen.1002157

**Published:** 2011-07-21

**Authors:** Michelle L. Churchman, Ignasi Roig, Maria Jasin, Scott Keeney, Charles J. Sherr

**Affiliations:** 1 Howard Hughes Medical Institute, St. Jude Children's Research Hospital, Memphis, Tennessee, United States of America; 2 Department of Genetics and Tumor Cell Biology, St. Jude Children's Research Hospital, Memphis, Tennessee, United States of America; 3 Molecular Biology Program, Memorial Sloan-Kettering Cancer Center, New York, New York, United States of America; 4 Cytology and Histology Unit, Department of Cell Biology, Physiology, and Immunology, Universitat Autonoma de Barcelona, Barcelona, Spain; 5 Developmental Biology Program, Memorial Sloan-Kettering Cancer Center, New York, New York, United States of America; Cornell University, United States of America

## Abstract

The mammalian *Cdkn2a* (*Ink4a-Arf*) locus encodes two tumor suppressor proteins (p16^Ink4a^ and p19^Arf^) that respectively enforce the anti-proliferative functions of the retinoblastoma protein (Rb) and the p53 transcription factor in response to oncogenic stress. Although p19^Arf^ is not normally detected in tissues of young adult mice, a notable exception occurs in the male germ line, where *Arf* is expressed in spermatogonia, but not in meiotic spermatocytes arising from them. Unlike other contexts in which the induction of *Arf* potently inhibits cell proliferation, expression of p19^Arf^ in spermatogonia does not interfere with mitotic cell division. Instead, inactivation of *Arf* triggers germ cell–autonomous, p53-dependent apoptosis of primary spermatocytes in late meiotic prophase, resulting in reduced sperm production. *Arf* deficiency also causes premature, elevated, and persistent accumulation of the phosphorylated histone variant H2AX, reduces numbers of chromosome-associated complexes of Rad51 and Dmc1 recombinases during meiotic prophase, and yields incompletely synapsed autosomes during pachynema. Inactivation of *Ink4a* increases the fraction of spermatogonia in S-phase and restores sperm numbers in *Ink4a-Arf* doubly deficient mice but does not abrogate γ-H2AX accumulation in spermatocytes or p53-dependent apoptosis resulting from *Arf* inactivation. Thus, as opposed to its canonical role as a tumor suppressor in inducing p53-dependent senescence or apoptosis, *Arf* expression in spermatogonia instead initiates a salutary feed-forward program that prevents p53-dependent apoptosis, contributing to the survival of meiotic male germ cells.

## Author Summary

The intimately linked *Arf* and *Ink4a* genes, encoded in part by overlapping reading frames within the *Cdkn2a* locus, are induced by oncogenic stress, activating the p53 and Rb tumor suppressors, respectively, to inhibit proliferation of incipient cancer cells. As such, expression of the p19^Arf^ and p16^Ink4a^ proteins is undetected in most normal mouse tissues. However, p19^Arf^ is physiologically expressed in mitotically dividing spermatogonia, the progenitor cells that differentiate to form meiotic spermatocytes in which *Arf* expression is extinguished. We show that, instead of provoking cell cycle arrest or death, *Arf* expression in spermatogonia facilitates survival of their meiotic progeny, ensuring production of normal numbers of mature sperm. When *Arf* is ablated, meiotic defects ensue, along with p53-dependent cell death of spermatocytes, indicating an unexpected role of p53 in monitoring meiotic progression. Surprisingly, it is the absence of p19^Arf^ rather than its induction that enforces p53 expression in this setting. Co-inactivation of *Ink4a* compensates for *Arf* loss by fueling proliferation of spermatogonial progenitors, but does not correct meiotic defects triggered by *Arf* loss. Although the *Arf* and *Ink4a* tumor suppressors are expected to restrain cellular self-renewal, *Arf* plays an unexpected role in male germ cells by facilitating their proper meiotic progression.

## Introduction

The *Cdkn2a*-*Cdkn2b* gene cluster (also designated *Ink4-Arf*) encodes two polypeptide inhibitors (p16^Ink4a^ and p15^Ink4b^) of cyclin D-dependent kinases (Cdk4 and Cdk6), as well as a third protein (p19^Arf^) that antagonizes the Mdm2 ubiquitin E3 ligase to activate p53 [1]. Although the *Ink4a* and *Ink4b* genes likely arose through gene duplication, the structure of the *Ink4-Arf* gene cluster is highly unusual, as major portions of the p16^Ink4a^ and p19^Arf^ proteins are encoded by alternative reading frames of a shared exon [2]. Induction of p16^Ink4a^ and p15^Ink4b^ prevents the phosphorylation of the retinoblastoma protein (Rb), thereby maintaining Rb in its growth suppressive state and preventing entry into the DNA synthetic (S) phase of the cell division cycle. In contrast, p19^Arf^ expression elicits a p53-dependent transcription program that either enforces cell cycle arrest or triggers apoptosis, depending on cell type, physiologic setting, and collateral modulating signals [1]. The *Ink4-Arf* genes prevent cell proliferation by implementing Rb- and p53-dependent programs that enforce cellular senescence and inhibit tissue regeneration as animals age, but their intimate genetic linkage facilitates their coordinate repression in embryonic and adult tissue stem cells, thereby allowing self-renewal [3], [4]. Deleterious growth-promoting stimuli conveyed by activated oncogenes induce *Ink4-Arf* gene expression and engage both p53 and Rb to counter untoward cellular proliferation. Not surprisingly, bi-allelic deletion of the *Ink4-Arf* gene cluster abrogates this form of tumor suppression and is one of the more frequent events in human cancer.

Despite its canonical role as an inducer of p53 in response to oncogene signaling, *Arf* also has p53-independent tumor suppressive activity. Deletion of *Arf* together with *Mdm2* and *p53* expands the spectrum and decreases the latency of cancers that spontaneously arise in mice lacking *p53*, *p53* and *Mdm2*, or *Arf* alone [5]. Although highly basic p19^Arf^ (∼20% arginine) has been reported to physically interact with more than 25 different proteins other than Mdm2, the role of p19^Arf^, if any, in regulating the functions of these putative “target” proteins remains controversial [6]. Indeed, numerous reports that p19^Arf^ regulates such diverse processes as ribosomal biosynthesis, transcription, DNA repair, apoptosis and autophagy in a p53-independent manner have generally relied on experiments performed with cultured cells but have not been buttressed by more extensive *in vivo* analyses.

Although the *Ink4-Arf* locus is not detectably expressed under most normal physiologic conditions, eye and male germ cell development provide notable exceptions [7]. *Arf* is required for early postnatal regression of the hyaloid vasculature in the vitreous, so that *Arf*-null mice form a retrolenticular mass predominantly composed of pericytes; the abnormal accumulation of these cells disrupts the retina and lens and leads to blindness [8]. *Arf* inactivation also results in a significant reduction of sperm production through as yet poorly defined mechanisms, although young male mice remain fertile [9]. In contrast, *Arf*-null females have no discernable reproductive defects.

Spermatogenesis involves a stereotyped sequence of mitotic and meiotic divisions followed by sperm differentiation [10]. In mice, male germ cell progenitors (gonocytes) renew in the testis between days 1–7 postpartum (P1–P7) and generate spermatogonia that line the basement membranes of developing seminiferous tubules [11], [12]. At P7–P10, spermatogonia divide to form preleptotene spermatocytes that detach from the basement membrane, are displaced toward the tubular lumen, and enter meiosis-I as primary leptotene spermatocytes. During the extended prophase of meiosis-I, homologous pairs of maternal and paternal chromosomes align to form synaptonemal complexes and exchange genetic information through homologous recombination [13]. Meiosis-I is completed by P18, and is followed rapidly by meiosis-II, and by spermiogenesis (P19–P35), after which the first mature spermatozoa enter the epididymis. As spermatogenesis continues throughout life, spermatogonia within mature seminiferous tubules remain localized on the peripheral tubular basement membrane, whereas spermatocytes, spermatids, and mature sperm are arranged in a sequential order from the periphery towards the lumen [10].

Intriguingly, p19^Arf^ is transiently expressed in mitotically dividing spermatogonia, but not in the meiotic cells that arise from them [9]. Here, we provide genetic evidence demonstrating that *Arf* expression initiates a germ cell autonomous program that protects meiotic spermatocytes from undergoing p53-dependent elimination. This physiologic function of p19^Arf^ directly contrasts with its role as a tumor suppressor in inducing p53.

## Results

### *Arf* Is Expressed in Mitotically Dividing Spermatogonia

**Figure 1:**
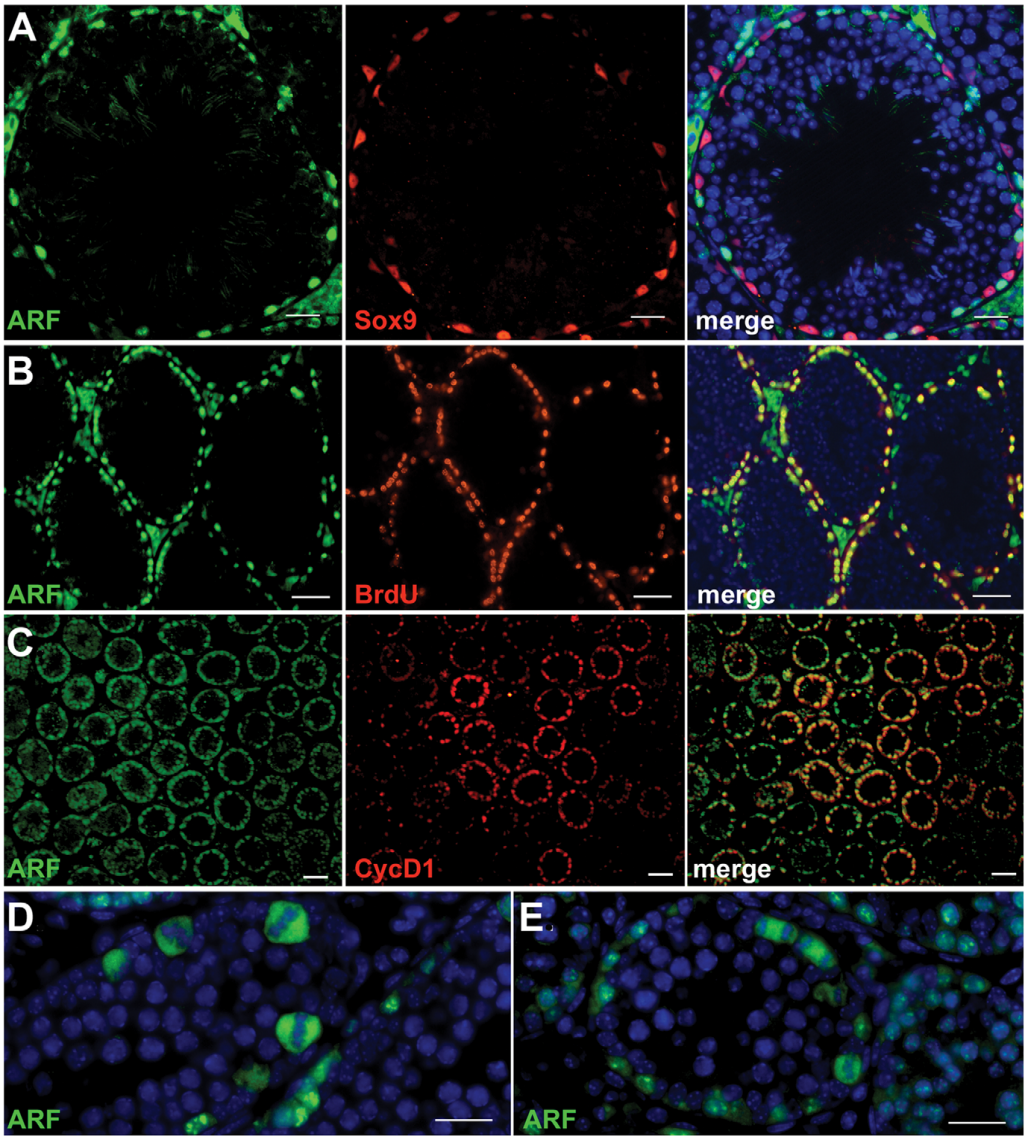
Arf protein expression in mitotically dividing spermatogonia. Protein expression in sections of seminiferous tubules were determined by immunofluorescence analysis. (A) p19^Arf^ (green, left panel) is expressed in spermatogonia that intervene between Sox9-expressing Sertoli cells (red, middle panel) in the seminiferous tubules of adult 4 month-old mice. The right panel shows merged images documenting no overlap in expression of the two proteins. Unlabeled cells within the lumina of the tubules are visualized with DAPI. (B) After a 2 hour *in vivo* pulse of bromodeoxyuridine (BrdU) in adult 5 month old mice, p19^Arf^ expression (green, left panel) was revealed in spermatogonia that had incorporated BrdU (red, middle panel). The right panel shows merged images documenting co-expression of both markers in many spermatogonia at the tubular periphery (yellow). (C) In seminiferous tubules from P12 mice, cells expressing p19^Arf^ (green, left panel) co-express cyclin D1 (red, middle panel), a protein expressed in actively cycling spermatogonia; a merged image is shown at the right. (D, E) Spermatogonia within the seminiferous tubules of P15 mice express p19^Arf^ (green) during mitosis. Examples of p19^Arf^ expression during metaphase (D) and telophase (E) are shown. DAPI (blue) highlights the nuclei of intratubular germ cells and somatic Leydig cells that occupy the intertubular space. Scale bars: (A, D, E) 50 µm; (B, C) 100 µm.

Lineage tracing experiments in the mouse previously revealed that all viable male germ cells are derived from spermatogonial progenitors in which transient *Arf* expression neither inhibits proliferation nor subsequent meiotic commitment [9]. Underscoring these findings, expression of p19^Arf^ in young adult mice is observed in all types of spermatogonia, but not in Sox9-expressing Sertoli cells on the tubular basement membrane or in DAPI-stained intratubular spermatocytes, spermatids, or sperm (Figure 1A). The fact that p19^Arf^ is not detected in cells that have detached from the basement membrane implies that *Arf* expression is extinguished at or near the primary spermatocyte stage of germ cell differentiation. Consistent with this interpretation, the Arf protein does not co-localize with Dmc1 [9], a meiotic recombinase expressed in leptotene spermatocytes. In the mature testis, spermatogenesis occurs in waves along the length of the seminiferous tubules, so that cross sections capture tubules in which dividing spermatogonia are in synchronous phases of the cell cycle. When five month-old mice injected intraperitoneally with BrdU were sacrificed two hours later, dual immunofluorescence analysis revealed that many cells on the tubular basement membrane that had synthesized DNA also expressed p19^Arf^ (Figure 1B). Similarly, at P12 when the number of mitotically cycling progenitors exceeds those of more differentiated germ cells, p19^Arf^ was co-expressed with cyclin D1, a G1 phase marker of proliferating spermatogonia [14] (Figure 1C), and strikingly, was detected during all stages of mitosis (Figure 1D, 1E). Therefore, in spermatogonia, p19^Arf^ is expressed throughout the cell division cycle without interfering with proliferation.

### *Arf* Deficiency Compromises Sperm Production, But Is Compensated by *Ink4a* Inactivation

**Figure 2:**
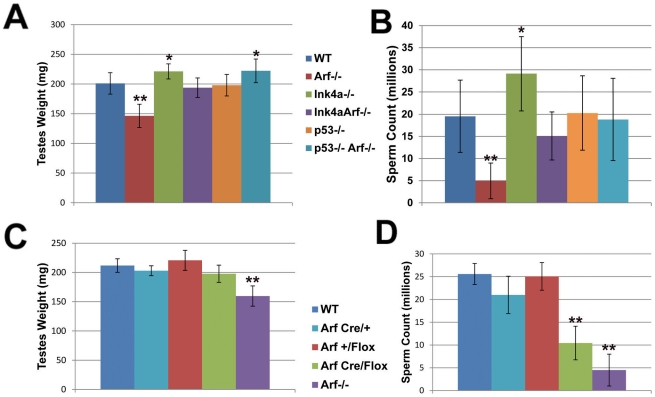
Decreased sperm production in *Arf*-null mice is compensated by loss of *Ink4a* or *p53*. (A, C) Testes were dissected from adult (2–6 month old) mice of the indicated genotypes and weighed as pairs. (B, D) Caudal epididymides were collected from corresponding mice, and recovered sperm were enumerated using a hemocytometer. Relative testes weights (A) and sperm counts (B) are reduced in *Arf*-null males but increased in *Ink4a*-null mice. *Ink4a-Arf* double-null and *p53* ^−/−^;* Arf* ^−/−^ double null mice exhibit increased testes weights (A) and sperm counts (B). While testes weights (C) are not significantly reduced in *Arf* ^Cre/Flox^ males, reduced sperm counts (D) mimic the *Arf* loss-of-function phenotype. [N = 20–32 mice (A, B) and 5 mice (C, D)]. Bars represent standard deviations from the mean. P values were determined using a Student's t-test (*p<0.001, **p<0.0001) and designate significant differences from the wild type genotype.

Total body weights of age-matched wild-type, *Arf*-null, *Ink4a*-null, and *Ink4a-Arf* double-null mice are equivalent, but testis weights of *Arf*-null animals were reduced relative to those of wild-type controls (Figure 2A), and this was associated with a significant reduction in numbers of mature sperm by the time *Arf*-null mice were two months old (Figure 2B). Nonetheless, young *Arf*-null males remain fertile, and despite the widespread use of independently derived *Arf*-null strains by us and others, there is no suggestion that young fertile males produce reduced litter sizes. Hence, defects in spermatogenesis were not previously appreciated.

**Figure 3:**
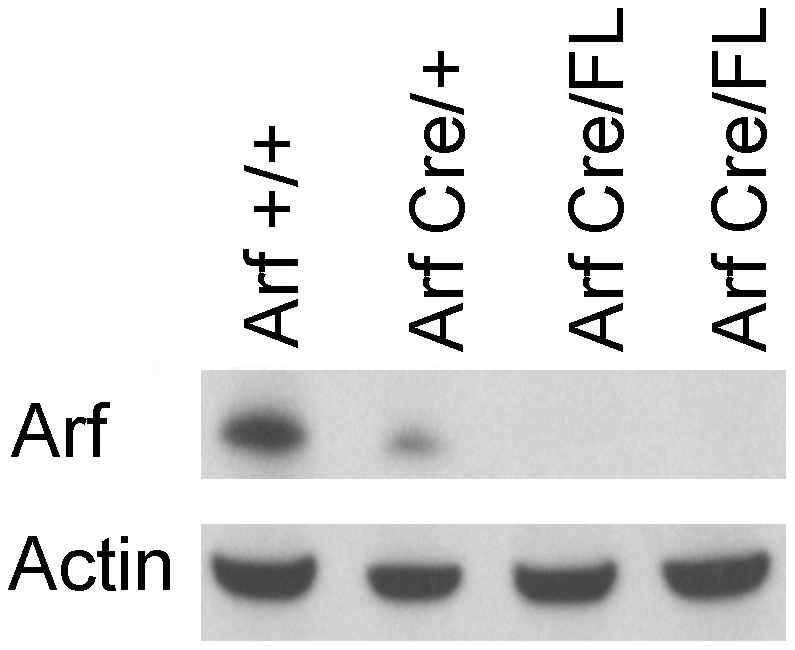
Loss of expression of p19^Arf^ in *Arf* ^Cre/Flox^ testes. Immunoblotting analysis was performed using an antibody against p19^Arf^. Whole testis lysates were prepared from two month old mice of the indicated genotypes. Immunoblotting with an antibody to actin was used to control for equal protein loading per lane.

Knock-in of a cDNA encoding Cre recombinase in place of the first *Arf* exon creates a functionally null *Arf* allele that expresses Cre in lieu of p19^Arf^ under the control of the *Arf* promoter. Crossing *Arf* ^Cre/+^ females to homozygous males containing *Arf* alleles flanked by LoxP sites (“floxed” alleles) specifically results in the inactivation of *Arf* function in the testis of compound heterozygous *Arf* ^Cre/Flox^ male offspring. Although penetrance of Cre expression is not complete, more than 90% of spermatogonia in the seminiferous tubules of P21 mice had no detectable anti-p19^Arf^ fluorescence signals [9]. Overall, while p19^Arf^ was detected in the testes of haplo-insufficient *Arf* ^Cre/+^ mice, any residual levels of the protein in *Arf* ^Cre/Flox^ testes were too low to be detected by immunoblotting analysis (representative data illustrated in Figure 3), confirming significant Cre-mediated *Arf* deletion in this setting. We therefore used this “targeted” deletion approach to compare the *Arf* loss-of-function phenotypes of *Arf* ^Cre/Flox^ males with those of *Arf^−/−^* males. Analysis of testis weights revealed no differences between those of *Arf* ^Cre/Flox^ mice and wild-type controls (Figure 2C). However, the sperm counts of *Arf* ^Cre/Flox^ animals were reduced to levels approaching those of *Arf* ^−/−^ males (Figure 2D). Notably, the *Arf-Cre* or *Arf-Flox* alleles alone had no significant effects in limiting sperm production unless coexpressed in compound heterozygotes. Therefore, tissue-restricted effects of *Arf* inactivation independently recapitulated those seen in mice that completely lack *Arf* function.

**Figure 4:**
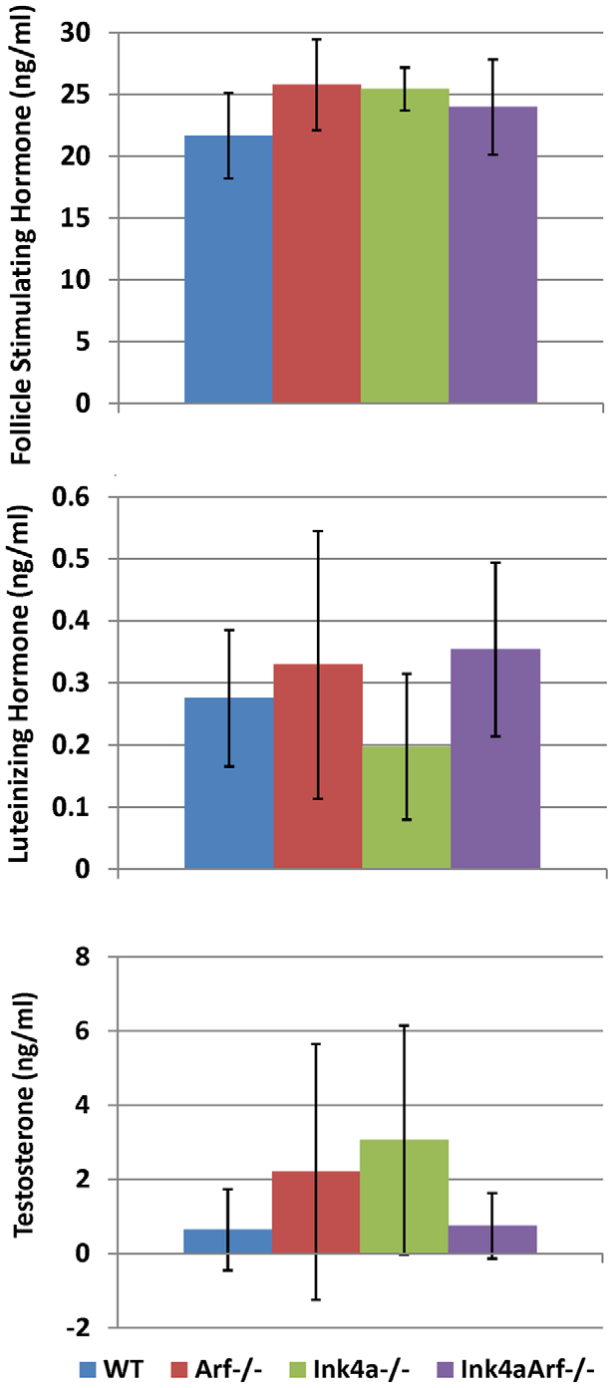
Hormone levels in mice of different genotypes. Blood was collected in the afternoon from 15 two to four month-old male mice of each indicated genotype, and sera were analyzed for hormone content. P values calculated using a Student's t-test indicated no significant differences between samples taken from mice of different genotypes. Wide day-to-day and even hour-to-hour fluctuations in plasma testosterone levels yield largely unpredictable changes of as much as 30-fold between individual samples, as determined by radioimmunoassay [19]. Error bars indicate standard deviations from the mean.

Hormone signaling networks are involved in the proper control of spermatogenesis. Key regulatory gonadotrophins include luteinizing hormone (LH) and follicle-stimulating hormone (FSH) secreted by the anterior pituitary gland, and testosterone produced by testicular interstitial Leydig cells. The considerable day-to-day and even hour-to-hour variation over a 30-fold range in plasma testosterone levels in age-matched mice of a single strain precluded accurate measurements of strain-specific differences, even in a relatively large sample size (Figure 4) [15]. Importantly, however, no discernable defects in pituitary or Leydig cell development have been observed in *Arf-*null, *Ink4a-*null, *or* doubly-deficient mice, and no significant differences were observed in the ranges of serum FSH and LH among all genotypes examined (Figure 4). These findings suggest that spermatogenesis defects in *Arf*-deficient mice are not a secondary consequence of hormonal imbalances.

**Figure 5:**
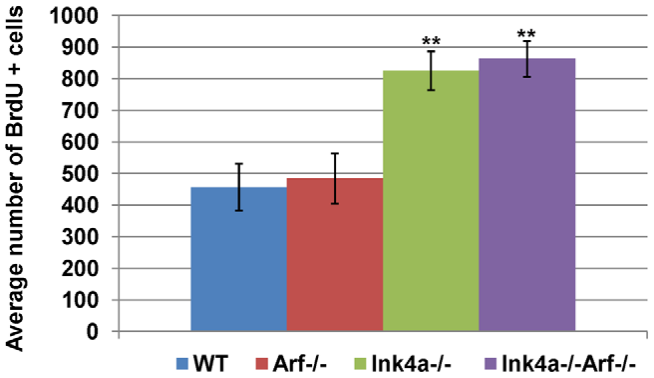
Increased frequency of BrdU-incorporating spermatogonia in *Ink4a*-null mice. Quantification of BrdU-positive spermatogonia in five month-old wild-type, *Arf*-null, *Ink4*a-null, and doubly *Ink4a* and *Arf*-null mice was determined two hours after intraperitoneal BrdU administration. BrdU-labeled cells were scored in 100 tubules in testis sections from seven different mice. Error bars indicate standard deviations from the mean. ** p<0.0001 vs wild-type by Student's t-test.

Unlike *Arf*-null males, those lacking functional *Ink4a* instead exhibit increased testis weights and produce higher numbers of sperm than wild type mice (Figure 2A and 2B). Cdk4, the major target of p16^Ink4a^ protein inhibition in the adult testis, is expressed at maximal levels at the earlier stages of spermatogenesis, where spermatogonia undergoing mitotic cell divisions predominate [16], [17], and *Cdk4* inactivation compromises male fertility [18], [19]. We therefore quantified the *in vivo* incorporation of BrdU in spermatogonia of young adult wild-type, *Arf*-null, *Ink4a*-null, and *Ink4a-Arf*-null mice by counting stained cells that had entered S phase during a two-hour pulse. The S phase fractions of wild-type and *Arf*-null spermatogonia did not differ from each other (Figure 5), implying that the failure of *Arf*-null mice to produce normal numbers of sperm reflects a loss of meiotic cells or their immediate progeny rather than spermatogonia. In contrast, we observed a significant two-fold increase (p<0.0001, Student's t-test) in the relative number of S phase spermatogonia from both strains that lack *Ink4a* (Figure 5). Looking only at testis weights and sperm counts in *Ink4a-Arf* double-null animals, *Ink4a* inactivation appears to compensate for loss of *Arf* function (Figure 2A and 2B), presumably by fueling the production of a greater number of mitotic progenitors. Together, the consequences of these two independent loss-of-function effects rebalance testis size and sperm output in the doubly null strain. In this sense, these two “tumor suppressor” genes play opposing physiologic roles in male germ cell development.

### *Arf* Inactivation Leads to p53-Dependent Apoptosis in Primary Spermatocytes

**Figure 6:**
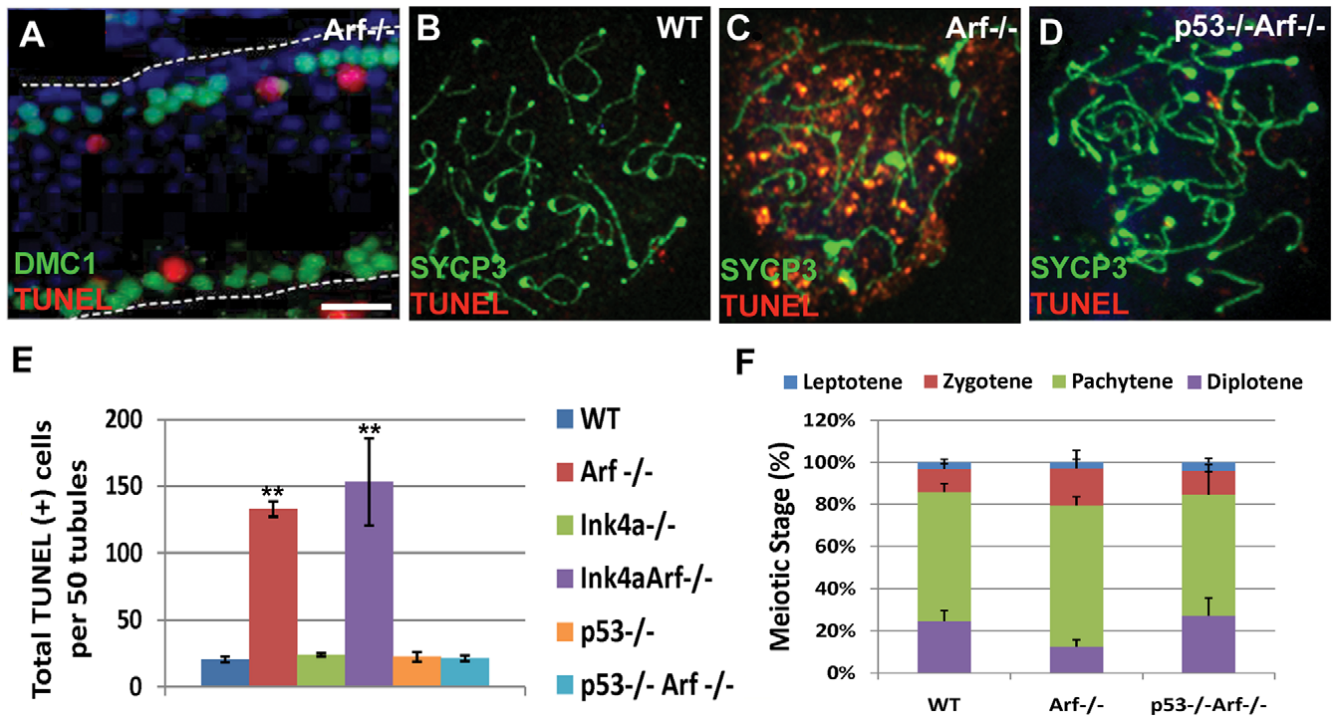
*Arf* deficiency leads to increased apoptosis of primary spermatocytes. (A) During spermatogenesis, germ cells move intraluminally as they differentiate, initially yielding primary meiotic spermatocytes (immunostained for Dmc1, green) that have detached from the basement membrane (represented by a dashed line). Dmc1-positive cells are visualized in a longitudinal section of a tubule from a two month-old *Arf*-null mouse. Spermatocytes within the tubular lumen are TUNEL-positive (red). DAPI (blue) marks the nuclei of all developing germ cells within the tubule. Neither DAPI-positive spermatogonia residing on the basement membrane nor more mature intraluminal cells express Dmc1 or are TUNEL-positive. (B–D) Surface spread spermatocytes from three month old wild-type (B), *Arf^−/−^* (C), and *p53* ^−/−^;* Arf* ^−/−^ (D) mice were immunostained for SYCP3 (green) and assayed for TUNEL (red). Although pachytene and diplotene spermatocytes from *Arf^−/−^* mice were TUNEL-positive, few such cells were identified in spreads from age-matched wildtype or *p53^−/−^*;* Arf^−/−^* mice. (E) TUNEL assays were performed on testis sections from 2–3 month old mice of the indicated genotypes. The total numbers of TUNEL-positive cells were enumerated in 50 tubules within three different sections taken from 3 different mice of each genotype. (F) One hundred surface spread spermatocytes (n = 3 mice) were categorized into the four phases of prophase I based on SYCP3 and γ-H2AX immunostaining patterns, as described in Materials and Methods. Errors bars represent standard deviations from the mean. ** p<0.0001 and *p<0.003 vs wild-type levels by Student's t-test.

Testes from two month-old *Arf*-null mice exhibited a significant increase in the numbers of apoptotic (TUNEL-positive) cells when compared to age-matched wild-type controls (Figure 6A and 6E). The vast majority of apoptotic cells are spermatocytes as judged by the topological relation of TUNEL-positive cells to the expression of the meiosis-specific strand-exchange protein Dmc1, which is expressed during early prophase-I (Figure 6A). Notably, however, intratubular TUNEL-positive cells were not stained with antibodies to Dmc1, implying that *Arf*-null cells die during a later stage of germ cell development after Dmc1 expression is greatly diminished. To examine this issue further, we conducted TUNEL staining of meiotic chromosome spreads. Characteristic stages of prophase during meiosis-I can be marked by staining chromosomes with antibodies to synaptonemal complex proteins, such as the axial element component SYCP3 [20], and by several ancillary criteria (see Materials and Methods). Unlike pachytene cells from wild-type mice, those from the *Arf*-null strain exhibited considerable TUNEL staining (Figure 6C) with a concomitant reduction in the fraction of *Arf*-null diplotene spermatocytes (Figure 6F) that correlated with decreased sperm production (Figure 2B). Inactivation of *Ink4a* alone did not trigger spermatocyte apoptosis nor limit apoptosis in the *Arf*-null background (Figure 6E) reinforcing the conclusion that the two closely linked genes play fundamentally different roles within the male germline.

**Figure 7:**
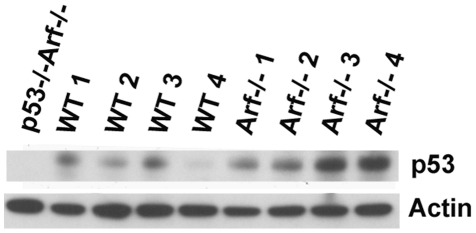
Levels of p53 detected in wild-type and *Arf*-null testis. Immunoblotting analysis was performed using whole testis lysates from four representative 3–4 month-old mice of each genotype. Actin was used as a loading control.

*Arf* ^−/−^; *p53* ^−/−^ doubly-deficient males are even more susceptible to spontaneous tumor development than mice lacking either *Arf* or *p53* alone [5]. However, the young tumor-free males produce sufficient viable sperm to remain fertile. Inactivation of *p53* restored testis weights and sperm production in *Arf-*null males (Figure 2A and 2B), prevented the apoptotic elimination of *Arf*-null germ cells (Figure 6D and 6E), and restored the number of diplotene spermatocytes (Figure 6F). Accordingly, higher levels of p53 were detected in whole testis lysates from *Arf*-null mice as compared to those in wild-type mice (Figure 7). Thus, in direct contrast to the role of p19^Arf^ in triggering a p53 response following abnormal hyperproliferative stress in somatic cells, it is instead the absence of *Arf* expression in spermatogonial progenitors that impairs the fidelity of meiotic progression and ultimately leads to p53-dependent elimination of *Arf*-null primary spermatocytes.

### Inappropriate γ-H2AX Accumulation in *Arf*-Null Germ Cells

**Figure 8:**
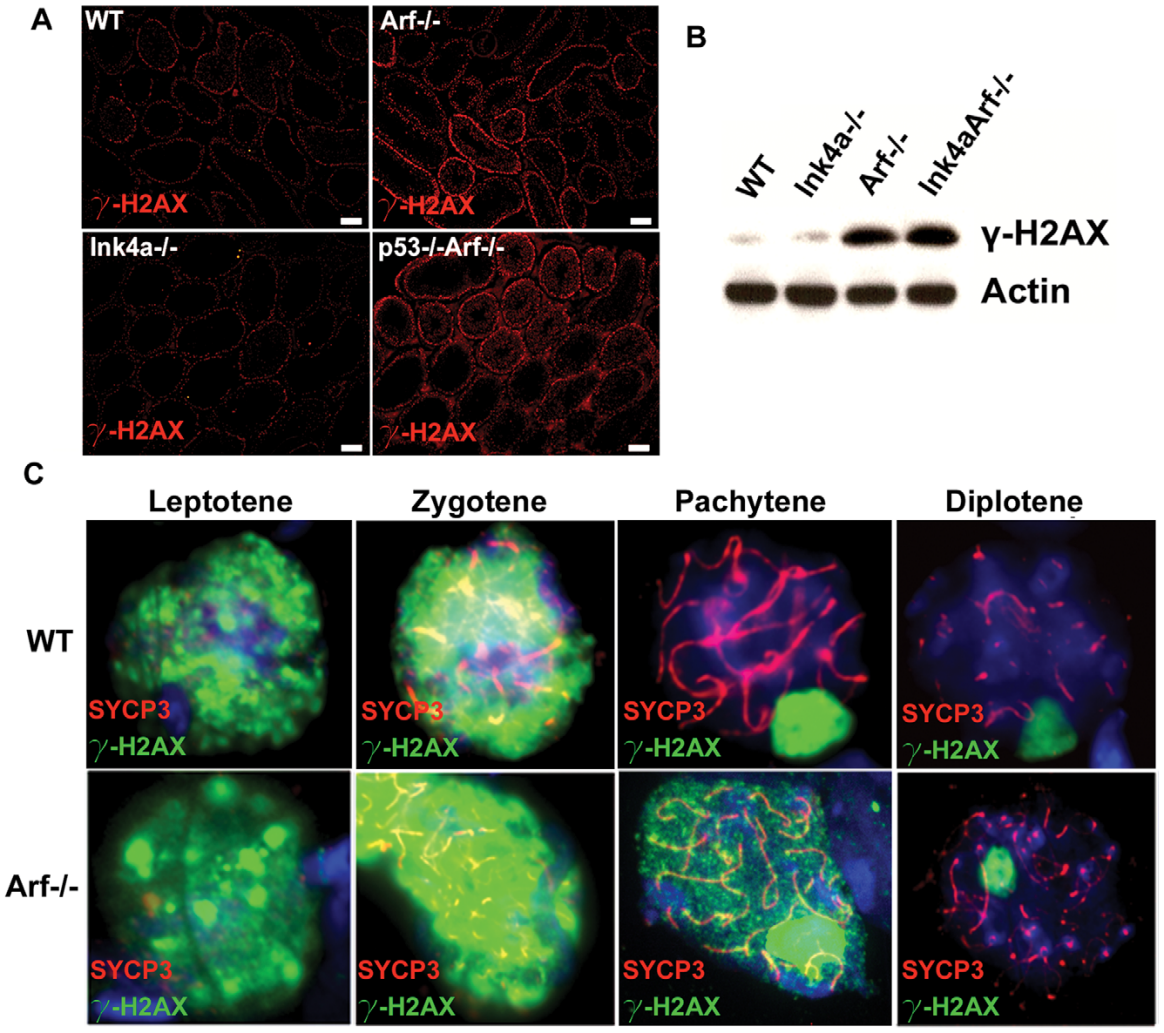
*Arf* deficiency provokes elevated and persistent γ-H2AX foci. (A) Testis sections from 3 month-old mice of the indicated genotypes were immunostained for γ-H2AX, as visualized here at low magnification to demonstrate overall relative intensities of staining. The brightest γ-H2AX-positive cells are primary spermatocytes. (B) Immunoblotting analysis of whole testis lysates from 3 month-old mice showing an accumulation of γ-H2AX in *Arf*-deficient testis. (C) Surface spread spermatocytes from three month old mice were immunostained for SYCP3 (red), γ-H2AX (green), and with DAPI to visualize nuclei (blue). Representative spreads from each stage of prophase I are shown for wild type (top) and *Arf*-null (bottom) strains, demonstrating persistence of autosome-associated γ-H2AX in pachytene spermatocytes from *Arf*-deficient mice. Staining of sex bodies persists throughout prophase in both genotypes. Images were captured with the same exposure times using a Zeiss Axioscope fluorescence microscope (A) or Intelligent Imaging Innovations Marianas spinning disc confocal microscope (C). Scale bars: (A) 100 µm.

**Figure 9:**
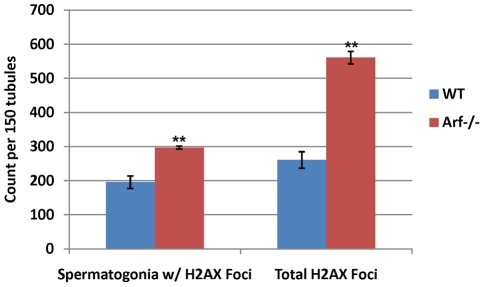
*Arf* deficiency leads to increased γ-H2AX foci in spermatogonia. The number of A and intermediate-type spermatogonia with γ-H2AX foci in 150 seminiferous tubules and the total number of foci contained within these cells were quantified in wild-type and *Arf*-null mice at P17. Enumeration of γ-H2AX foci within 150 tubules in each of three different sections from each genotype was performed at high magnification for foci limited to spermatogonia distinguished by morphology and position along the basement membrane. P values were determined using a Student's t- test; **p value<0.0001 vs wild-type. Error bars indicate standard deviations from the mean.

Histone H2AX is phosphorylated at serine-139 in response to DNA strand breaks caused by ionizing radiation [21], UV irradiation [22], replication stress [23], [24], failure of nucleotide excision repair [25], and at the leptotene stage of meiosis prior to synaptonemal complex formation [26]. H2AX phosphorylation also occurs in spermatocytes in a DNA damage-independent manner during formation of the sex body, a heterochromatic sub-nuclear domain encompassing the nonhomologous parts of the X and Y chromosomes [27]. Remarkably, staining of testis sections and immunoblotting of whole testis lysates revealed a profound increase in global γ-H2AX levels when *Arf* was inactivated (Figure 8A and 8B). Again, inactivation of *Ink4a* neither recapitulated nor ameliorated this *Arf*-null defect (Figure 8A and 8B). Germ cells at the periphery of *Arf*-null seminiferous tubules exhibited the greatest increase in γ-H2AX staining, suggesting that more immature cells were the ones most affected (Figure 8A). Microscopic quantification revealed that the number of γ-H2AX-positive spermatogonia, as well as the number of γ-H2AX foci per cell, were increased ∼2-fold when *Arf* was inactivated (Figure 9), but the greatest increase in γ-H2AX staining was observed in primary *Arf*-null spermatocytes (Figure 8A and 8B; see below). The increased γ-H2AX in meiotic cells is especially striking because these cells do not normally express p19^Arf^ when the gene is present (Figure 1).

Meiotic double-strand DNA breaks are induced by the Spo11 transesterase and its accessory factors, which are loaded onto chromatin during the final pre-meiotic S-phase [13]. Autosomal γ-H2AX staining is normally observed during the leptotene and zygotene phases of meiosis-I, which are the early stages at which chromatids undergo DNA scission as a prelude to homologous recombination. In contrast, γ-H2AX foci are not normally detected by early pachytene (except in the sex body) once homologous synapsis is complete (Figure 8C, top panels) [28]. Chromosome spreads from meiotic primary spermatocytes from *Arf*-null males revealed that 150 of 382 individually enumerated pachytene cells (39%) exhibited persistent autosomal γ-H2AX foci in addition to normal sex body staining, whereas very few such cells (6.7%) were detected at the diplotene stage (Figure 8C, bottom panels). It could be that disappearance of γ-H2AX is delayed until diplonema, or that cells with aberrantly elevated γ-H2AX are preferentially eliminated. The latter interpretation is supported by the prophase I apoptosis and depletion of diplotene cells observed in *Arf*-null mice (Figure 6). Therefore, in the absence of *Arf*, γ-H2AX accumulates to higher levels than normal starting in the least mature spermatogonia, continuing into meiotic prophase I, and persisting past the time when it would normally disappear from autosomes. Although inactivation of *p53* suppresses the increased apoptosis of *Arf*-null spermatocytes, γ-H2AX persists in cells lacking both of these genes (Figure 8A, lower right panel). In meiotic chromosome spreads from *p53*; *Arf* double-null mice, 20.5% (34 of 166) of diplotene spermatocytes display persistant autosomal γ-H2AX immunostaining as compared to 6.7% (5 of 74) of singly *Arf*-null and less than 1% (1 of 149) of wild-type diplotene spermatocytes. These data underscore the fact that the accumulation of γ-H2AX in *Arf*-null spermatocytes is p53-independent, whereas the elimination of defective spermatocytes that retain γ-H2AX inappropriately is p53-dependent.

### Additional Meiotic Prophase Defects in *Arf*-Null Primary Spermatocytes

**Figure 10:**
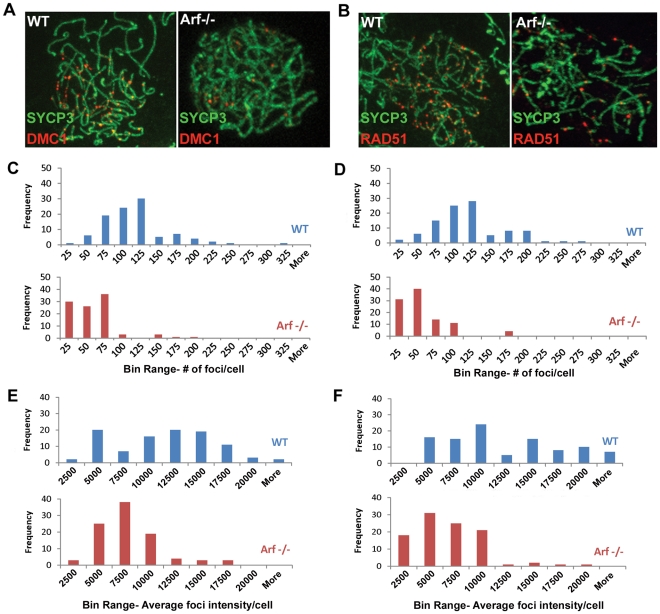
Diminished Dmc1/Rad51 focus formation in *Arf*-deficient spermatocytes. (A, B) Surface spread spermatocytes from three month old WT (left panels) and *Arf*-null (right panels) mice were immunostained for SCP3 (A, B; green) and Dmc1 (A; red) or Rad51 (B; red). Images were captured with the same exposure time using a Marianas spinning disc confocal microscope. (C, D) Histograms showing the distribution of the number of Dmc1 (C) and Rad51 (D) foci found in wild-type (blue bars) and *Arf*-null (red bars) spermatocytes. Foci were enumerated from one hundred zygotene spermatocytes immunostained for SYCP3 and either Dmc1 (C) or Rad51 (D) using Slidebook 5.0 SDC software. Actual values of foci per cell are plotted within bin ranges to display the distribution of frequencies. (E, F) Histograms showing the distribution of average intensities of Dmc1 (C) and Rad51 (D) foci found in wild-type (blue bars) and *Arf*-null (red bars) spermatocytes analyzed in panels C and D.

DNA double-strand breaks induced early in prophase I by Spo11 serve as substrates for the strand exchange proteins Rad51 and meiosis-specific Dmc1, which are required for double strand break repair during homologous recombination. Foci of staining using antibodies to Dmc1 (Figure 10A) and Rad51 (Figure 10B) were readily observed in zygotene spermatocytes from wild-type mice (left panels) but were fewer and less prominent in their *Arf*-null counterparts (right panels). The number and average fluorescence intensities of foci in 100 zygotene cells of each genotype were determined using commercial imaging software. In wild-type zygotene spermatocytes, the frequency of Dmc1 and Rad51 foci peaked at 100–125 per cell (Figure 10C and 10D, respectively, blue bars) and exhibited a broad distribution of relative intensities over a ∼10-fold range (Figure 10E and 10F, blue bars). In contrast, both the number and intensities of Dmc1/Rad51 foci were significantly reduced in *Arf*-null cells (average number of foci ± S.D.: 103±46 Dmc1 foci in wild-type vs. 44±34 in *Arf* ^−/−^; 108±45 Rad51 foci in wild-type vs. 45±33 in *Arf* ^−/−^; average relative intensities ± S.D.: 10239±4959 Dmc1 foci in wild-type vs. 6611±3005 in *Arf* ^−/−^; 10980±5783 Rad51 foci in wild-type vs. 5637±3340 in *Arf* ^−/−^ N = 100, p<0.0001, Student's t-test; Figure 10C–10F). An accumulation of *Arf*-null spermatocytes in zygonema (Figure 6F) suggests that there may be a delay at this stage before progression to pachytene.

**Figure 11:**
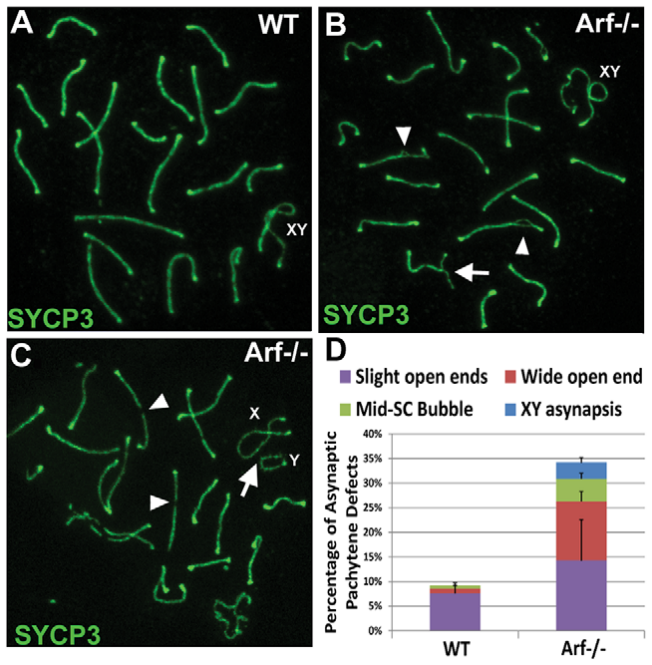
*Arf*-null pachytene spermatocytes exhibit synaptic defects. (A–C) Staining of surface spread spermatocytes for SYCP3 marks the lateral elements of bivalents. (A) In wildtype pachytene spermatocytes, fully synapsed autosomal bivalents are observed, as judged by continuous SYCP3 staining along the axes. (B–C) Representative synaptic defects in *Arf*-null pachytene spermatocytes include (B) unsynapsed ends (arrow) and interstitial asynaptic “bubbles” (arrow heads), and (C) asynapsis of the X and Y chromosomes (arrow). Synapsed X and Y chromosomes are shown in (A, B). Synaptonemal complexes with segmental disruption of SYCP3 staining were more frequently observed (p = 0.051) in *Arf*-null cells (C, arrowheads) versus their wild-type counterparts (191 versus 53 such segments in 300 pachytene chromosomal spreads from each genotype). (D) Quantification of synaptic defects observed in wild-type and *Arf*-null pachytene spermatocytes. “Wide open” ends are illustrated in (B); less extensive asynapsis at telomeres was categorized as “slight” open ends. 100 pachytene spermatocytes from each of three mice of different genotypes yielded highly significant differences in overall defects (p = 0.0017 by Student's t-test). Error bars indicate standard deviation from the mean.

Pachytene cells are normally characterized by well developed synaptonemal complexes that stretch the length of autosome axes and by knob-like accumulation of SYCP3 at telomeres (Figure 11A). However, 34% of *Arf*-null cells exhibited defects in synapsis (quantified in Figure 11D), including forked terminal structures and interstitial bubbles on autosomes (Figure 11B) and complete asynapsis of sex chromosomes (arrow, Figure 11C). In addition, interrupted regions of SYCP3 staining (denoted by arrowheads in Figure 11C) were more frequently observed in meiotic chromosome spreads from *Arf*-null cells versus those in wild-type cells (191 versus 53 such segments, respectively, in 300 pachytene cells of each genotype). Because synapsis was complete in the majority of *Arf*-null pachytene cells, we could not distinguish whether the observed defects arose from regions in which synaptonemal complexes did not form at all, or where complexes had formed but subsequently disassembled. Taken together, *Arf*-deficiency results in a series of abnormalities during prophase I that include reduced loading of the Rad51 and Dmc1 recombinases, defects in synapsis, elevated and persistent γ-H2AX expression, and p53-dependent apoptosis, ultimately associated with diminished production of mature sperm.

## Discussion

With few exceptions, the *Arf* tumor suppressor is not expressed in normal tissues of healthy mice but is induced by abnormally sustained and elevated thresholds of proliferative signals, activating a p53 response that opposes the deleterious effects of oncogene activation. Notably, p53 responds to a much wider range of *Arf*-independent signal transduction cascades triggered by many other forms of cellular stress, including acute DNA damage, to which the *Arf* promoter does not respond [6]. By converging on p53, these different signaling pathways inhibit cell cycle progression or trigger apoptosis, acting to suppress tumor formation.

We now document a physiological role of *Arf* in mouse male germ cell development that is distinct from its tumor suppressive functions in key respects. First, *Arf* is expressed in spermatogonia, but not in the primary spermatocytes that arise from them. Expression of p19^Arf^ neither arrests spermatogonial mitotic progression nor triggers their p53-dependent apoptosis. However, the absence of *Arf* expression in spermatogonia leads to p53-dependent apoptosis of spermatocytes before they exit meiosis-I. The defect in spermatogenesis is germ cell autonomous and results in a significant reduction in sperm counts by the time *Arf*-null mice are two months old, although residual sperm production maintains fertility in young males. Thus, expression of *Arf* in mitotic progenitor cells enhances the survival of their meiotic progeny in which *Arf* expression is normally extinguished. These features indicate that *Arf* expression initiates a salutary, feed-forward program that facilitates meiotic progression. Indeed, although *Arf* and *Ink4a* are widely viewed to convey tumor suppressive functions that coordinate the activities of the p53 and Rb signaling “pathways,” inactivation of *Arf* and *Ink4a* in the testes leads to opposing outcomes. Disruption of *Ink4a* increases the mitotic activity of spermatogonial progenitors to enhance sperm output and, in this respect, compensates for *Arf* loss of function without eliminating the cellular defects that arise in the *Arf*-null setting. In short, loss of *Ink4a* increases the spermatogonial pool size, but without *Arf* expression, spermatocytes undergo increased apoptosis, returning the number of mature sperm to normal levels.

Homologous recombination during meiosis exchanges genetic information between maternally and paternally derived chromosomes and also guides proper segregation of chromosome pairs to maintain correct chromosome numbers in gametes [13]. During meiosis, in contrast to mitotically diving cells, homologous chromosomes are favored over sister chromatids as templates for recombinational DNA repair. Double-strand DNA breaks are formed by the topoisomerase-II-related transesterase Spo11. This process activates the Atm kinase and leads to phosphorylation of the H2AX histone variant near sites of strand breakage during early prophase I. Binding of the RecA family strand exchange proteins, Rad51 and meiosis-specific Dmc1, to Spo11-induced DNA ends generates filaments that search for and invade homologous duplex DNA molecules, leading to pairing of homologous chromosomes. Loading of Rad51 and Dmc1 is normally reversed by early pachytene when chromosomes are fully synapsed, after which γ-H2AX foci are no longer detected.

In the *Arf*-null setting, a modest but significant increase in γ-H2AX staining was first detected in the least mature spermatogonia, and primary spermatocytes displayed accentuated signals that persisted inappropriately into the pachytene stage. *Arf*-null cells also formed fewer Dmc1/Rad51 foci at zygotene and exhibited focal regions of asynapsis at pachytene. Aberrant *Arf*-null spermatocytes underwent apoptosis at pachytene, resulting in the emergence of fewer diplotene cells and a significant reduction in sperm output. Importantly, *Arf* ^−/−^; *p53* ^−/−^ double-null pachytene cells also exhibited persistent γ-H2AX staining, but these cells escaped elimination. Thus, apoptosis was p53-dependent, but aberrant γ-H2AX accumulation was not.

Although the underlying mechanisms remain unknown, we consider here two plausible interpretations of this apoptotic arrest. First, it may be that reduced Rad51/Dmc1 focus formation and persistent γ-H2AX staining in *Arf*-null male germ cells connote a defect in DNA repair that then activates p53 through *Arf*-independent but Atm/Atr-dependent signaling pathways. In this scenario, Spo11-induced DSBs would form at normal levels but Rad51/Dmc1 loading would be impaired such that some DNA damage would persist into pachytene. This might conceivably involve the p53-independent ability of p19^Arf^ to promote the sumoylation of numerous target proteins by inhibiting the SUMO2/3 protease Senp3 [29]–[31]. SUMO2/3 accumulates at sites of DNA damage in mammalian cells [32], [33], and various aspects of DNA repair are regulated by the SUMO conjugation pathway [34]. There is fragmentary evidence that absence of p19^Arf^ compromises nucleotide excision repair in cultured cells [35], [36] raising the possibility that *Arf* may play an as yet undefined role in promoting homologous recombination. All meiotic mutants that cannot properly synapse homologous chromosomes arrest during pachytene [37], and accompanying defects in sex body formation and failure to properly silence transcription of the sex chromosomes during prophase is itself sufficient to eliminate pachytene cells [27], [38]. However, spermatocytes can also undergo apoptosis in direct response to unrepaired Spo11-induced breaks even if sex body formation is normal [27], [39]. Where tested, spermatocyte apoptosis in meiotic mutants with chromosome synapsis errors has been found to be p53-independent [40]–[42]. Moreover, Spo11-dependent activated phospho-p53 can be transiently detected from leptonema and zygonema in wild-type male mice, and in *Drosophila*, p53 activity is prolonged in cells defective for meiotic repair [43]. Thus, it remains a formal possibility that meiotic recombination defects can trigger p53-dependent apoptosis.

A second, alternative interpretation rests on the idea that the earlier and less profound accumulation of γ-H2AX in *Arf*-null spermatogonia might be a symptom of an underlying defect affecting chromatin structure or Atm/Atr signaling. The appearance of γ-H2AX reflects chromatin modifications that flank sites of DNA damage rather than strand breaks themselves, so the kinetics of γ-H2AX formation and dissolution do not necessarily coincide with the appearance and repair of DNA damage [44], [45]. Moreover, aberrant Atm/Atr signaling is itself sufficient to activate p53, whether triggered by DNA breaks or not [46]. Thus, it may be that *Arf* deficiency causes inappropriate Atm/Atr signaling that provokes p53-dependent apoptosis in a DNA damage-independent manner. In this view, the observed meiotic prophase defects in *Arf*-null spermatocytes may possibly be a separate downstream consequence of this earlier anomaly, and may not be the cause of apoptosis. Regardless of which interpretation is correct, it is important to note that our findings provide strong evidence that p53-dependent monitoring promotes proper meiotic maturation, in addition to the previously documented p53-independent pathway(s). Whatever the underlying mechanisms, the role of *Arf* in male germ cell development contrasts with the general paradigm of p19^Arf^ acting as an activator of p53. Instead, it is the absence of *Arf* in spermatogonia that consequently leads to p53-dependent apoptosis of spermatocytes.

## Materials and Methods

### Ethics Statement

No human or non-human primates were studied. All animal work with mice was performed under established guidelines and supervision by the St. Jude Children's Research Hospital's Institutional Animal Care and Use Committee (IACUC), as required by the United States Animal Welfare Act and NIH policy to ensure proper care and use of laboratory animals for research. Experiments were undertaken in an accredited facility of the Association for Assessment of Laboratory Animal Care under the supervision of trained veterinary personnel and in strict compliance with Howard Hughes Medical Institute, St. Jude Children's Research Hospital, and NIH institutional guidelines. The latter include detailed protocol submission and review of all animal care, monitoring, and experimental procedures prior to initiation of any experiments. Ongoing protocols for animal research not necessitating interim amendments are minimally subjected to annual review by the IACUC. All persons involved in the use of animals have read and understand all implications of pertinent protocols, have received training in the execution of relevant animal-related procedures prior to participation in the protocol, and have participated in educational or training programs deemed necessary by the IACUC or the Animal Resources Center personnel. Studies reported herein did not unnecessarily duplicate previous research, and were undertaken only because suitable non-animal models were unavailable. The number of animals used was consistent with good statistical design. Anesthesia, analgesia and tranquilization were used to relieve pain and distress in accordance with the IACUC recommendations.

### Mouse Strains

*Arf*-null [47], *Arf*-GFP [7], *Arf*-Flox and *Arf*-Cre mice [9] were generated in the Sherr laboratory. Mouse strains deficient for *Ink4a* [48] and *Ink4a-Arf* [49] were generously provided by R.A. DePinho (Dana Farber Cancer Center). All genetically engineered mice were backcrossed nine or more times onto a C57Bl/6 background to create isogenic strains. C57Bl/6 mice deficient for *p53* were purchased from Jackson Laboratories (Stock Number 2101). *Arf* ^GFP/GFP^ mice were crossed to *p53* ^+/−^ mice, and compound heterozygotes were interbred to generate *Arf* ^GFP/GFP^;*p53* ^−/−^ mice functionally null for both genes. *Arf* ^Cre/+^ females were interbred with *Arf* ^Flox/Flox^ males to generate *Arf* ^Cre/Flox^ mice.

### Phenotypic Characterization of Mouse Testes and Sperm Count Analysis

Caudal epididymides were harvested before dissection of the testes. For each male mouse, two cauda were minced into 1 ml of Dulbecco's modified Eagle's medium (DMEM) containing 25 mM HEPES buffer (pH 7.5) and 4 mg/ml bovine serum albumin and incubated at 37°C for 20 minutes. Suspensions of sperm were fixed at a 1∶25 dilution in 10% formalin and counted on a hemocytometer. All sperm counts were performed between 1:00–3:00 PM. Dissected testes were weighed in pairs.

### Immunofluorescence of Testes Sections

Mice were euthanized by CO_2_ asphyxiation, and testes were removed and fixed overnight at 4°C in 4% paraformaldehyde followed by saturation in 30% sucrose at 4°C overnight. Tissues were embedded in TBS Tissue Freezing Medium (Fisher Scientific, Pittsburg PA), and sliced with a HM500M Cryostat (Microm International, Walldorf, Germany) into 10 µm sections. Fixed and frozen samples were sectioned and subjected to antigen retrieval in 0.1 M Na citrate buffer, pH 6.0, followed by one hour incubation at room temperature in a blocking solution of 10% normal goat serum (NGS), 0.1% Triton-X 100 in phosphate-buffered saline (PBS), and then by overnight incubation at 4°C in primary antibodies diluted in 3% NGS, 0.1% Triton-X 100 in PBS. Antibodies were directed to p19^Arf^ [rat monoclonal immunoglobulin 5C3-1 [50], Sox9 (Millipore AB5535, 1∶1000), BrdU (Santa Cruz sc32323, 1∶100), cyclin D1 (Santa Cruz 72-13G, 1∶750), Dmc1 (Santa Cruz H-100, 1∶750), γ-H2AX (Cell Signaling 2577, 1∶200), and SUMO2/3 (Cell Signaling 18H8, 1∶300). Slides were washed three times in PBS, and then incubated for 1 hour at room temperature in 3% NGS, 0.1% Triton-X 100 in PBS containing the relevant secondary antibodies conjugated to Ig-Alexa Fluor 555 or Ig-Alexa Fluor 488 (1∶500 dilutions; Invitrogen). Slides were washed three times in PBS and mounted with Vectashield (Vector Labs) containing 4′-6-diamidino-2-phenylindol (DAPI). TUNEL assays were performed using an *in situ* cell death detection kit (TMR red, Roche) following the manufacturer's protocol. Images of tissue sections were photographed using a Zeiss Axioscope fluorescence microscope and assembled using Zeiss Axiovision software.

### Analysis and Staging of Meiotic Spreads

Testes were decapsulated and minced in 5 ml of DMEM per testis and transferred to a 15 ml Falcon tube. After further dissociation of the tubules by pipeting up and down, large pieces were allowed to settle to the bottom of the tube by gravity for 10 minutes on ice. One ml of the supernatant, containing a suspension of spermatocytes, was transferred to a 1.5 ml Eppendorf tube and centrifuged for five minutes at 5800× g. The pellet was resuspended in 40 µl of a 0.1 M sucrose solution, and 20 µl of spermatocyte suspension was applied evenly to a slide containing a thin layer of 1% paraformaldehyde (pH 9.2) containing 0.1% Triton X-100. Slides were allowed to dry for two hours at room temperature in a closed humidity chamber before rinsing in Photo-flo (Kodak 1464510, diluted 1∶250 in doubly distilled H_2_O) and air dried at room temperature. For immunofluorescence, slides were incubated in PTBG (0.2% bovine serum albumin, 0.2% gelatin, 0.05% Tween 20 in PBS) for 10 minutes with shaking. Primary antibodies were diluted in PTBG, applied to the slide, and covered with parafilm before incubation overnight at 4°C in a humidity chamber. Antibodies were directed to SYCP3 (Santa Cruz G-3, 1∶500) to mark the synaptonemal axial element [20], to γ-H2AX (Cell Signaling 2577, 1∶500) to identify sex body formation and sites of DNA damage, and to Rad51 (Calbiochem Ab-1, 1∶500) and Dmc1 (Santa Cruz H-100, 1∶750) to demonstrate formation of complexes required for DNA strand exchange during homologous recombination. Slides were washed three times in PTBG at room temperature for 3 minutes with shaking. Secondary antibodies, also diluted in PTBG, were applied to slides which were covered with parafilm and incubated at 37°C for one hour in a humidity chamber. Slides were washed three times in PTBG for 3 minute intervals in the dark with shaking and mounted with Vectashield (Vector Labs) containing DAPI. Surface spread spermatocytes were visualized by a Marianas spinning-disc confocal microscope, and images were assembled and analyzed using Slidebook 5.0 SDC software (Intelligent Imaging Innovations, Denver CO). Meiotic spreads from three adult mice (age three months) were analyzed. One hundred spermatocytes were scored each from mouse.

Distinct staining patterns allow for classification of each stage of meiotic prophase [51], [52]. Leptotene cells were categorized by short stretches of axial elements accompanied by intense γ-H2AX staining throughout the nucleus and the absence of a distinct sex body. Zygotene cells also display intense γ-H2AX staining throughout the nucleus and lack a sex body, but can be distinguished by longer stretches of SYCP3 staining, some of which are synapsed. Pachytene cells have fully formed and synapsed axes that appear as thick, continuous SYCP3-stained threads, while displaying intense γ-H2AX staining only in the sex body. Dmc1 and Rad51 foci are normally present at leptotene and zygotene, and largely disappear by pachytene. Diplotene cells have γ-H2AX localized only to the sex body, but fully formed axes are desynapsing and chiasmata are visible.

### Immunoblotting

As previously described [53], detergent lysates were prepared, and protein concentration was quantified by bicinchoninic acid assay (Pierce). Samples (25–75 µg protein per lane) were electrophoretically separated on 4% to 12% Bis-Tris NuPAGE gels (Invitrogen), transferred to polyvinylidene fluoride membranes (Millipore), and detected using antibodies to γ-H2AX (Cell Signaling S139, 1∶500), p19^Arf^ (5C3-1; Bertwistle et al. 2004b), p53 (Cell Signaling 1C12, 1∶500), and actin (Santa Cruz C-11, 1∶500) to control for protein loading.
